# First Case of Splenic Abscess Parvimonas micra and Bacteremia Porphyromonas gingivalis Coinfection

**DOI:** 10.7759/cureus.28103

**Published:** 2022-08-17

**Authors:** Angela Ho, Bharati Duvapu, Ngoc Duong, Sandhyarani Samantara

**Affiliations:** 1 Medical School, Touro College of Osteopathic Medicine, Middletown, USA; 2 Internal Medicine, Putnam Hospital Center, Carmel, USA

**Keywords:** bacterial co-infection, bacteremia, splenic abscess, parvimonas micra, porphyromona gingivalis

## Abstract

We report a case of a 76-year-old Caucasian male with bacteremia caused by *Porphyromonas gingivalis* and splenic abscess caused by *Parvimonas micra.* This patient presented with nonspecific symptoms: fever, chills, body aches, and shortness of breath. He was treated with IV piperacillin-tazobactam that was later switched to ampicillin sodium/sulbactam sodium during his hospital course and underwent a splenectomy. He ultimately expired due to acute respiratory failure and cardiac arrest, secondary to post-surgical complications. To our knowledge, this is the first case of *P. micra* and *P. gingivalis* coinfection.

## Introduction

*Porphyromona gingivalis* is a gram-negative anaerobe that accounts for 10-15% of periodontal diseases in adults [1,2]. There has been one case of bacteremia due to *P. gingivalis *reported in the literature [3]. A patient with bacteremia will become symptomatic when immune response mechanisms fail or are overwhelmed, which can lead to many clinical spectrums [4]. More specifically, bacteremic older patients typically present with symptoms including but not limited to fever, chills, increased erythrocyte sedimentation rate, recently altered mental status, and leukopenia [5]. *Staphylococcus aureus* is the most common gram-positive associated bacteremia, while *Escherichia coli* is the most common gram-negative organism [4]. Broad-spectrum antibiotics including cephalosporin or beta-lactamase inhibitors are used to treat bacteremia. In addition, vancomycin can be added to cover resistant gram-positive organisms [4].

*Parvimonas micra* is a gram-positive anaerobic coccus that is typically found in the oral flora and is also often associated with periodontal diseases [6]. Risk factors for *P. micra* infection include various dental procedures and systemic disease (diabetes mellitus, corticosteroid treatment) [6]. Cobo et al. identified 31 cases of *P. micra *infection with the first documented case in 1986 [7]. The spine has been the reported preferred location of infection; however, *P. micra* has also been associated with heart valve infections and perirenal, hepatic, and intracranial abscesses [8-11]. The incidence of splenic abscesses is very low [12]. Symptoms of infection by *P. micra* are non-specific (fever, chills) and the choice of treatment has not yet been established [11]. 

We report a case of a 76-year-old man with splenic abscess caused by *P. micra* and bacteremia due to *P. gingivalis* treated successfully with splenectomy, vancomycin, piperacillin-tazobactam, and ampicillin sodium/sulbactam sodium.

## Case presentation

A 76-year-old-man with a past medical history of tobacco abuse, hyperlipidemia, stage 3 chronic kidney disease, and hypertension presented to the Emergency Department (ED) with shortness of breath, chills, fever, and body aches. The patient reported a maximum temperature of 102°F recorded at home. He also has a history of multiple dental cleanings, with a cleaning every three months, and the most recent cleaning a month ago. The patient was febrile (100.3°F), with a heart rate of 85 beats per minute, a respiratory rate of 21 breaths per minute, blood pressure of 128/70, and an oxygen saturation of 96%. Dental evaluation did not identify any periodontal source of infection. The differential diagnosis at this time included coronavirus disease 2019 (COVID-19), bronchitis, febrile illness, and viral infection. The COVID-19 test was negative, his x-ray results showed no acute pulmonary process, and EKG showed normal sinus rhythm (NSR); so, the patient was discharged home.

He returned to the hospital later that night with an additional complaint of sharp, constant abdominal pain localized to the left upper quadrant. On the physical exam, he was guarded, but did not have any rebound tenderness. He also presented with the same chills and fever as he did in the morning. The patient denied any prior history of abdominal surgeries. Lab values were significant for elevated troponin at 121 ng/L, with a normal sinus rhythm EKG reading, white blood cell count of 11.4 x 10^3^/µl, creatinine 2.07 mg/dL, blood urea nitrogen (BUN) 114 mg/dL, and lactate 2.4 mmol/L. Code sepsis was called, and blood cultures were collected. The patient became hypoxic and was put on a 2 L oxygen nasal cannula (NC). Throughout the stay, the patient’s oxygen requirements stayed around 2-3 L. 

**Figure 1:**
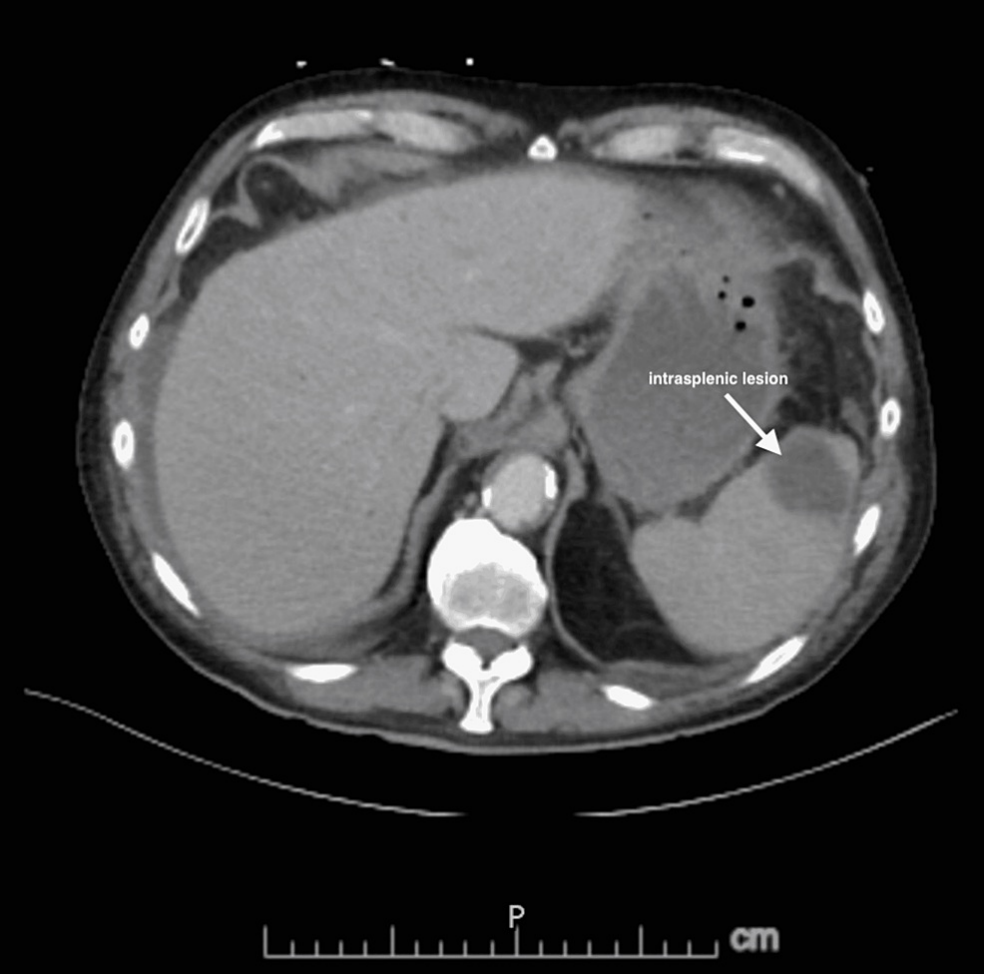
Abdominal CT scan showing an intrasplenic lesion.

Upon admission to the hospital, the patient received IV fluid boluses and antibiotics, including vancomycin 1000 mg, piperacillin-tazobactam 3.375 gm, and ampicillin sodium/sulbactam sodium 1.5 gm. The patient was stabilized. The patient was also receiving labetalol 200 mg, nifedipine 30 mg, and atorvastatin 4 mg for hyperlipidemia. His CT showed a small amount of free fluid in the abdomen, but no evidence of an abscess. An intrasplenic lesion was also noted, as well as extensive atherosclerotic changes in the abdominal aorta (Figure 1). The patient’s troponin and lactate levels were progressively trending downwards. His abdominal pain began to improve. However, the patient’s acute kidney injury still remained, possibly due to a multifactorial use including but not limited to the use of antibiotics including vancomycin, contrast imaging studies, sepsis, and fluid overload. Echocardiogram did not show any major cause for the volume overload. Transthoracic echocardiogram was negative for endocarditis.

The splenic lesion was examined via a fluid collection and subsequently drained percutaneously. This aspiration showed bloody purulent fluid and blood cultures were positive for growth of *P. gingivalis* and *P. micra *on hospital days nine and 10, respectively. Transesophageal echocardiogram showed right pleural effusion without cardiac vegetations, ruling out infective endocarditis. 

Once the patient was hemodynamically stable, he underwent a splenectomy. After his splenectomy, vancomycin was discontinued on hospital day 10, and treatment with piperacillin-tazobactam (Zosyn) was switched to ampicillin sodium/sulbactam sodium (Unasyn) on hospital day 15. On day 16, the patient developed emesis and subsequently acute respiratory distress syndrome (ARDS). He went into cardiac arrest, and was successfully resuscitated and intubated. EKG showed normal sinus rhythm, chest x-ray was significant for bibasilar atelectasis, and his renal function was worsening. The patient later had another cardiac arrest in the afternoon, from which he did not survive.

## Discussion

To our knowledge, this is the first case of splenic abscess formation by *P. micra*. It is well known that the oral cavity is composed of many microbiological organisms. According to Watanabe et al, *P. micra* can be found in periodontal disease, infective endocarditis, and intra abdominal abscess [9]. Of patients with *P. micra* infection, 75% presented with several risk factors including several dental procedures as seen in our patient who received multiple dental cleanings [6]. Despite the fact that dental evaluation did not show periodontal disease, the most likely source of the infection remains translocation of microorganism from oral cavity to GI tract. Further, infections caused by *P. micra* are rare and even less likely to form splenic abscesses. It may be difficult to initially diagnose due to its non-specific manifestations of symptoms. The diagnosis of splenic abscess caused by *P. micra* should be suspected in patients with frequent dental procedures as in this case. 

Treatment of *P. micra* has not been established; however, drainage of abscess and treatment with vancomycin, ceftriaxone, and metronidazole have been effective in treating this microorganism [10]. A previous case study showed that most *P. micra* infections are susceptible to penicillin, clindamycin, imipenem, and metronidazole, although metronidazole-resistant strains exist [13]. Our patient was treated with IV vancomycin, which was discontinued on hospital day 10 post-percutaneous splenic abscess aspiration and drainage. There is no gold standard for treating splenic abscesses [14]. Although less invasive procedures can be successful in treating splenic abscesses, there are some cases where splenectomy is the best treatment option. There are reports of people with splenic abscesses who did not get a splenectomy and eventually expired due to systemic complications [15]. Conservative treatment with *P. micra* can be risky as there is no set treatment for this microorganism. Thus, our patient underwent a splenectomy given the risk of conservative treatment failure. Splenic abscess has a high mortality rate; therefore, laparoscopic splenectomy is a promising alternative, with quicker recovery and shorter hospital stays [16]. 

*P. gingivalis* is an uncommon rare cause of bacteremia that has been associated with the development of septicemia causing hypotension, altered mental status, and hypovolemia from leaking capillaries [4]. Other organs can be affected such as the lungs and kidneys leading to ARDS and acute kidney injury as in this case [4]. Upon return to the ED, our patient developed hypoxic ARDS and acute kidney failure and required oxygen supplementation with 2L oxygen NC and IV fluid boluses. Any delay in the administration of antibiotics can lead to increased morbidity and mortality; thus bacteremia requires urgent treatment by appropriate antibiotics [4]. 

Treatment options for *P. gingivalis* infection can include tetracyclines, macrolides, clindamycin, beta lactam antibiotics, and metronidazole [15]. However, later studies have shown *P. gingivalis* recently has been highly resistant to clindamycin, metronidazole, and amoxicillin [16]. Our patient was initially being treated with IV Zosyn and switched to Unasyn on hospital day 15 after splenectomy for more targeted treatment. *P. gingivalis* is shown to be 100% sensitive to moxifloxacin and amoxicillin/clavulanic acid but has moderate susceptibilities to other antibiotics [17]. Therefore, Unasyn may be effective in treating this pathogen. 

During the patient’s postoperative recovery, he developed acute respiratory failure. The etiology of his respiratory decompensation is not entirely clear, but may be due to aspiration pneumonia given multiple episodes of emesis the night prior to the development of ARDS. Aspiration of gastric contents is the leading risk for ARDS [18]. He had two consecutive cardiac arrest events due to ongoing hypoxemia and metabolic acidosis. This pathogenic process was further complicated by his ongoing bacteremia infection and acute kidney injury that progressed to acute kidney necrosis, further worsening his condition. Ultimately, the combination of multi-organ decompensation led to his death.

## Conclusions

In summary, *P. micra* and *P. gingivalis* are rare causes of splenic abscess and bacteremia, respectively. It is also rare for these bacteria to become bloodborne. While most patients with systemic *P. micra* or *P. gingivalis* bacteria usually present with infective endocarditis, that was not the case for our patient. Unasyn may be effective in treating this coinfection; however, it is important to be cautious regarding postoperative complications following splenectomy. Future research on these two microbes is required to provide proper treatment.
